# A conceptual disease model for adult Pompe disease

**DOI:** 10.1186/s13023-015-0334-6

**Published:** 2015-09-15

**Authors:** Tim A. Kanters, W. Ken Redekop, Maureen P.M.H. Rutten-Van Mölken, Michelle E. Kruijshaar, Deniz Güngör, Ans T. van der Ploeg, Leona Hakkaart

**Affiliations:** Institute for Medical Technology Assessment, Department of Health Policy & Management, Erasmus University Rotterdam, BOX 1738, 3000DR Rotterdam, The Netherlands; Center for Lysosomal and Metabolic Diseases, Erasmus MC University Medical Center, Rotterdam, The Netherlands

**Keywords:** Orphan drugs, Conceptual model, Pompe disease

## Abstract

**Background:**

Studies in orphan diseases are, by nature, confronted with small patient populations, meaning that randomized controlled trials will have limited statistical power. In order to estimate the effectiveness of treatments in orphan diseases and extrapolate effects into the future, alternative models might be needed. The purpose of this study is to develop a conceptual disease model for Pompe disease in adults (an orphan disease). This conceptual model describes the associations between the most important levels of health concepts for Pompe disease in adults, from biological parameters via physiological parameters, symptoms and functional indicators to health perceptions and final health outcomes as measured in terms of health-related quality of life.

**Methods:**

The structure of the Wilson-Cleary health outcomes model was used as a blueprint, and filled with clinically relevant aspects for Pompe disease based on literature and expert opinion. Multiple observations per patient from a Dutch cohort study in untreated patients were used to quantify the relationships between the different levels of health concepts in the model by means of regression analyses.

**Results:**

Enzyme activity, muscle strength, respiratory function, fatigue, level of handicap, general health perceptions, mental and physical component scales and utility described the different levels of health concepts in the Wilson-Cleary model for Pompe disease. Regression analyses showed that functional status was affected by fatigue, muscle strength and respiratory function. Health perceptions were affected by handicap. In turn, self-reported quality of life was affected by health perceptions.

**Conclusions:**

We conceptualized a disease model that incorporated the mechanisms believed to be responsible for impaired quality of life in Pompe disease. The model provides a comprehensive overview of various aspects of Pompe disease in adults, which can be useful for both clinicians and policymakers to support their multi-faceted decision making.

**Electronic supplementary material:**

The online version of this article (doi:10.1186/s13023-015-0334-6) contains supplementary material, which is available to authorized users.

## Background

Research in rare diseases is complicated by several methodological difficulties, which especially arise from insufficient statistical power due to low patient numbers [1]. Despite these methodological difficulties, policy makers require information on the burden of the disease and the therapeutic value of orphan therapies to support their decisions on reimbursement [2]. This information is especially important because orphan therapies are often associated with considerable costs per patient, accumulating to up to €600,000 per patient per year for some orphan drugs [3].

In the absence of long-term follow-up data on sufficiently large numbers of patients – as is the case for many orphan diseases – conceptual models can be used to describe the disease and assess treatment benefits. Such models allow the extrapolation of outcomes based on relatively short-term follow-up data of a limited number of patients, by combining available data with known disease-specific correlations.

The conceptual model developed by Wilson and Cleary (1995) provides a conceptual framework. It describes the relationship between different aspects of the disease (from biological parameters to functioning), health perceptions and overall quality of life [4]. The Wilson-Cleary model consists of five distinct levels of health concepts: 1) biological and physiological factors, focusing on the function of cells, organs, and organ systems; 2) symptom status, the patient’s perception of an abnormal physical emotional or cognitive state, including, for instance, fatigue, worry and depression; 3) functional health, the ability of the individual to perform particular defined tasks; 4) general health perceptions, a patient’s subjective rating of an integrated concept of all health aspects; and 5) overall quality of life, the individual’s state of overall well-being. The model combines clinical and social science models by categorizing health outcomes into underlying health concepts and by identifying causal relationships between the different concepts. As such, the model describes a causal pathway from clinical outcome parameters to quality of life. The model can be used to estimate quality of life on the basis of patients’ clinical data.

Pompe disease is an inherited, metabolic orphan disease. It is caused by a deficiency of the enzyme alpha-glucosidase needed for the degradation of lysosomal glycogen, and leads to storage of glycogen in many tissues [5]. Pompe disease has a broad clinical spectrum. The severe, classic infantile form is accompanied by muscle and heart malfunctioning, leading to death within the first year of life [6–8]. The majority of Pompe patients (~80 %) present at adult ages with muscle weakness. Limb girdle and respiratory muscles are predominantly affected [8–10]. Eventually, most patients require ambulatory and respiratory support and life expectancy is decreased compared to the general population [11]. Enzyme replacement therapy (ERT) with alglucosidase alfa has proven to be effective in Pompe disease [12–15]. Treatment of adult patients with Pompe disease with ERT is lifelong and associated with annual costs of up to €382,000 per patient [3]. Such high treatment costs potentially translate in a high incremental cost-effectiveness ratio.

To support reimbursement decisions, information on the burden of disease in untreated patients is needed, as well as on the effect of treatment and its cost-effectiveness. Ultimately, a model to estimate the cost-effectiveness of a treatment requires information on survival and quality of life in both treated and untreated patients as well as on the costs of treatment.

The purpose of this study is to develop a conceptual model for Pompe disease in adults and statistically test it in untreated patients. The structure of the conceptual model was established by applying the concept of the Wilson-Cleary model to Pompe disease, describing the associations between biological and physiological variables, symptoms, functional status, general health perceptions and health-related quality of life for this disease. The model can be used to predict health outcomes and can also be the starting point of a cost-effectiveness model, if information about disease progression, treatment effects and costs are added to it.

## Methods

### Part 1: Application of the Wilson-Cleary model to Pompe disease

Modelling guidelines prescribe extensive consultations with clinical experts in the development of a model [16]. Therefore, variables were included in the draft conceptual model for Pompe disease based on clinical relevance, as derived from clinical expert opinion and review of the literature. The model was discussed with experts from the Dutch Center of expertise for Pompe disease repeatedly until consensus was reached on the draft model. Next, the selected variables were assigned to the different levels of health concepts of the Wilson-Cleary model.

### Part 2: Statistically testing of the relationships of the model

To come to a final conceptual model, the relationships between the different levels of health concepts of the draft conceptual model were quantified in the second part of the study. In line with modelling guidelines, which recommend that a conceptual model should not be driven by data availability [16], clinical plausibility rather than statistical significance of an association determined whether variables were included in the final model.

#### Study sample and data collection

The study population consisted of patients with a proven diagnosis of Pompe disease by enzyme analysis and/or mutation analysis. Data was collected between January 2005 and January 2011. During this period, 103 adult Pompe patients were followed at Erasmus Medical Center in Rotterdam, the Dutch national center of expertise for Pompe disease. Clinical data was collected during regular standardized follow-up examinations at the Erasmus Medical Center. Patient reported outcome data were obtained from the ongoing International Pompe Association (IPA) survey in which all Dutch patients participate [11] and a burden of illness study in Pompe disease in adults [17]. The dataset consisted of multiple observations per patient, obtained at irregular time intervals and with a variable number of observations per patient. Only data on adult patients who were untreated at the time of observation were included in the analyses. No other inclusion criteria were applied; meaning that all adult patients diagnosed with Pompe disease were eligible for inclusion in the study, from mild to severely affected patients. Only patients for which data was available on at least two adjacent levels of health concepts were included in the regression analyses. All patients provided informed consent and the studies were approved by the Medical Ethics Committee of Erasmus MC.

#### Statistical analyses

We explored the strength of the relationships depicted in the conceptual model by means of random effects linear regression analyses. The regressions estimate relationships for a cross sectional unit during a particular time. Explanatory variables and dependent variables are dated at the same point in time (e.g. functioning at t = 1 is used to model general health perceptions at t = 1) [18]. The regression analyses account for interdependence of multiple observations per patient. The analyses represent cross-sectional analyses on multiple observations per patient. A separate regression analysis was run for each relationship between levels of health concepts in the conceptual model. In total eight different sets of analyses were performed, shown as models I to VIII in the results section. The different levels of health concepts of the conceptual model were first used as dependent variables in the regression analyses and subsequently as explanatory variables in the model for the next level. Levels of health concepts in the model (i.e. biological and physiological variables, symptom status, functional status, health perceptions and quality of life) could entail multiple variables. For each regression model, the combined significance of all variables was tested by means of a Wald Chi square test. The number of patients and observations per patient varied in the different regression analyses. The significance level was set at *p* = 0.05. The percentage of variance explained by the model was assessed by the overall R^2^ of the regression models. Statistical analyses were performed using Stata version 13 (StataCorp, 2013).

## Results

### Part 1: Development of a conceptual model for Pompe disease based on the Wilson-Cleary model

#### Biological and physiological variables

The first level of health concepts of the Wilson-Cleary model essentially describes two aspects: biological factors and physiological factors. In the conceptual model for Pompe disease, these were disentangled to allow modeling of the relationship between the biological cause of Pompe disease and physiological variables. Reduced activity of the enzyme acid alpha alglucosidase is the biological cause of Pompe disease [5]. We therefore included enzyme activity (measured in fibroblasts as a percentage of enzyme activity of non-Pompe patients) in the model as a biological variable.

With respect to physiological aspects, experts proposed the use of skeletal muscle strength and respiratory function [8, 13, 19–21]. Skeletal muscle strength was assessed by manual muscle testing using the grading scale from 0 (no visible contraction) to 5 (normal strength) of the Medical Research Council (MRC). For selected muscle groups, the MRC scores were summed, and subsequently the MRC sum score was expressed as a percentage of the maximum possible score (as described in [22]). Respiratory function was measured using forced vital capacity (FVC) in sitting position. Results were expressed as a percentage of the predicted normal value derived from published data [23].

#### Symptom status

The second level of health concepts in the Wilson-Cleary model covers symptoms. Adult Pompe patients have been shown to experience fatigue [24, 25]. Shortness of breath is another symptom frequently experienced by Pompe patients [26]. Therefore, fatigue and shortness of breath were proposed to reflect symptom status in the draft conceptual model. Fatigue was assessed using the Fatigue Severity Scale (FSS), which examines the self-reported effects of fatigue on daily functioning [27]. The scale ranges from 1 (no fatigue) to 7 (extremely fatigued), with a score of 4 or higher indicating that a patient is fatigued. Shortness of breath was not assessed in this patient population. Since no data were available on shortness of breath, a direct relationship between physiological variables (MRC sum score and FVC) and functional status was included in the model explaining functional status, in addition to FSS.

#### Functional status

The third level of health concepts in the Wilson-Cleary model is functional status. The self-reported nine item Rotterdam Handicap Scale (RHS) was considered to be a suitable measure of functional status. The RHS measures participation in daily life of patients and comprises 9 items that can be scored from 1 (unable to fulfill the task or activity) to 4 (complete fulfillment of the task or activity) [28]. The RHS score ranges from 9 to 36, with lower levels of handicap being associated with higher RHS scores. Hagemans et al. (2007) applied the RHS in Pompe disease and showed that daily life was substantially affected by the disease, especially with respect to job/study performance and ability to fulfill domestic tasks [29].

#### General health perceptions

The fourth level of health concepts of the Wilson-Cleary model includes general health perceptions, characterized by Wilson-Cleary as 1) an integration of the health concepts discussed above plus others (such as mental health) and 2) being a subjective rating. Two patients can objectively have the same health state but may have a very different perception of their health, potentially because of differences in factors like coping or self-efficacy. Health perceptions can be measured using the EQ-5D Visual Analogue Scale (EQ-5D VAS) [30]. Patients indicate their perceived health status on a thermometer ranging from 0 (worst perceived health status) to 100 (best perceived health status).

#### Quality of life

The final level of health concepts of the Wilson-Cleary model is quality of life (QoL). Previous studies have shown that QoL is lower in patients with Pompe disease compared to the general Dutch population [17, 21, 31]. QoL can be measured using the Mental Component Scale (MCS) and Physical Component Scale (PCS) of the Short-Form 36 (SF-36) [32] as well as in utilities derived from the EQ-5D. Utilities range from 0 (representing death) to 1 (representing perfect health). The SF-36 is a validated instrument to describe a patient’s health status [33]. MCS and PCS can be constructed from the eight distinct domains of health derived from the SF-36. U.S. norm based scores were used to compute PCS and MCS [32]. Two studies showed that PCS was significantly lower in patients with Pompe disease compared to the general population, but found that MCS was not affected by the disease [21, 31]. The SF-36 reflects a patient’s self-reported health status, whereas a utility provides a valuation of health based on the perceived importance of different domains by the general public. In our study, utilities were derived from the widely used EQ-5D [30], a validated tool to measure quality of life. The Dutch tariff was used to calculate utilities [34].

#### Characteristics of the individual and the environment

We hypothesized that age, gender and disease duration (time since diagnosis) could be used as individual patient characteristics potentially affecting all levels of health concepts in the conceptual Pompe disease model, except for the biological level [20, 21, 35–38].

### Part 2: Quantification of the relationships in the draft conceptual model

#### Study population

For 79 patients (77 % of the total Dutch adult Pompe population) who did not receive ERT data was available on at least two adjacent levels of health concepts. Half of the population was female, average age at baseline was 49.6 years and baseline disease duration since diagnosis was 8.0 years (Table 1). The maximum follow-up period was 5.4 years while the median follow-up was 1.1 years. The maximum number of observations per patient was 12. The median number of observations per patient was two and ten patients had only a single observation.

**Table 1:** Patient characteristics at baseline

Number of patients	N	Mean	Min	Max
Age (years)	79	49.3	23.0	72.6
Gender (female)	79	50.6 %		
Duration since diagnosis (years)	79	8.0	0.0	27.5
Enzyme activity (% of normal)	79	12.4	0.5	19.9
Muscle strength (% predicted)	64	82.1	48.3	100.0
Respiratory function (% predicted)	63	76.1	11.3	123.4
Fatigue Severity Scale	61	5.6	2	7
Rotterdam Handicap Scale	64	27.4	13.5	36.0
Visual Analogue Scale	49	65.0	30.0	95.0
Mental Component Scale	60	53.8	24.2	74.0
Physical Component Scale	60	35.4	17.6	53.3
EQ-5D Utility	50	0.736	0.201	1.000

#### Quantitative evaluation of the draft conceptual model

Table 2 presents the results of the multivariate regression models. Models I (MRC sum score as the dependent variable) and II (FVC) describe physiological variables, model III (FSS) represents symptom status, model IV (RHS) represents functional status, model V represents health perceptions (EQ-5D VAS), and models VI, VII and VIII (MCS, PCS and Utility respectively) describe quality of life.

**Table 2:** Regression coefficients based on a random effects model

Level	Physiological variables		Symptom status	Functional status	Health perceptions	Health related quality of life		
Model	I MRC	II FVC	III FSS	IV RHS	V EQ-5D VAS	VI MCS	VII PCS	VIII Utility
Constant	**83.452**	**70.526**	**8.737**	**16.989**	−9.313	**49.990**	**24.526**	**0.530**
Age	−0.069	−0.144	0.011	**−0.096**	0.126	−0.016	−0.046	0.001
Female	−0.028	**17.994**	0.307	−0.957	4.673	−1.282	−0.402	0.010
Disease duration	**−0.563**	−0.346	**−0.077**	−0.052	**0.653**	0.063	−0.086	−0.001
Enzyme activity	0.370	−0.431	0.007	−0.048	0.004	−0.359	−0.069	−0.006
Muscle strength			−0.031	**0.189**				
Respiratory function			−0.011	**0.075**				
Fatigue Severity Scale				**−0.578**				
Rotterdam Handicap Scale					**2.030**			
Visual Analogue Scale						**0.157**	**0.223**	**0.004**
patients	78	72	61	61	73	73	73	71
observations	249	257	160	158	154	152	152	161
R^2^ (overall)	0.105	0.075	0.322	0.623	0.343	0.183	0.293	0.354
Wald test (p-value)	**<0.001**	**0.001**	**0.002**	**<0.001**	**<0.001**	**0.005**	**<0.001**	**<0.001**

Figures in bold indicate significance at 5 % level; Enzyme activity, muscle strength and respiratory function were measured as a percentage of normal values, Visual Analogue Scale, Mental Component Scale and Physical Component Scale were measured on a scale from 0–100, Fatigue Severity Scale was measured on a scale from 1–7, Rotterdam Handicap Scale was measured on a scale from 0–36, Utility was measured on a scale from 0–1

#### Models predicting the physiological level of health concepts (models I and II)

The MRC sum score was negatively associated with disease duration (*p* < 0.001). One additional year with the disease was associated with a 0.6 % point reduction in the MRC sum score. FVC was not associated with disease duration (*p* = 0.190). The MRC sum score and FVC were not related to age (*p* = 0.411 and *p* = 0.459, respectively). However, if disease duration was not included in the model, age was significantly related to MRC sum score (0.240 percentage points decrease per year; *p* = 0.005), but age was not significantly related to FVC (*p* = 0.135). Female patients had a higher FVC (by 18.0 percentage points) than males (*p* = 0.001) but there was no difference between males and females in MRC sum scores (*p* = 0.990). Only baseline observations were available for enzyme activity and were assumed constant for later observations. Baseline enzyme activity was not significantly related to muscle strength (*p* = 0.215) or respiratory function (*p* = 0.556).

#### Model predicting the symptom status level of health concepts (model III)

FSS was negatively associated with disease duration (*p* < 0.001). One additional year with the disease was associated with a 0.08 point decrease of FSS. FSS was not associated with age (*p* = 0.389), gender (*p* = 0.382), enzyme activity (*p* = 0.893), or FVC. FSS was associated with the MRC sum score only at a 10 % significance level (*p* = .070).

No data on shortness of breath were available for this population.

#### Model predicting the functional status level of health concepts (model IV)

RHS was significantly affected by FSS (*p* < 0.003). Every 1 point increase in FSS was associated with a decrease of 0.5 in handicap scores. Furthermore, RHS was significantly affected by MRC sum score and FVC (both *p* < 0.001). Every 1 % point increase in MRC sum score (FVC) was associated with an improvement of 0.2 (0.1) in handicap scores. Age also had a significant association with handicap scores in that older patients had poorer scores than younger patients (0.1 points per year increase in age; *p* < 0.004). Gender, disease duration and enzyme activity were not significantly associated with RHS scores.

#### Model predicting the general health perceptions level of health concepts (model V)

The EQ-5D VAS was significantly associated with Rotterdam Handicap Scale (*p* < 0.001). An increase of 1 point in RHS score translated in an increase in EQ-5D VAS of 2 points. Disease duration was also positively associated to EQ-5D VAS (*p* = 0.002). Age, gender and enzyme activity were not related to EQ-5D VAS.

#### Models predicting the health related quality of life level of health concepts (models VI, VII and VIII)

MCS was not significantly associated with age, disease duration, gender, enzyme activity. MCS was significantly associated with the EQ-5D VAS (*p* < 0.0001); a 1 point increase on the EQ-5D VAS would lead to an increase in MCS of 0.2.

PCS was associated with EQ-5D VAS (*p* < 0.001); a 1 point increase in EQ-5D VAS would result in an increase of the PCS with 0.2 points. PCS was not affected by age, gender, disease duration and enzyme activity.

Utility was significantly and positively associated with EQ-5D VAS (*p* < 0.001). An increase with 1 point on EQ-5D VAS leads to an increase in utility of 0.004. Utility was not associated to age, gender, disease duration or enzyme activity.

**Fig. 1:**
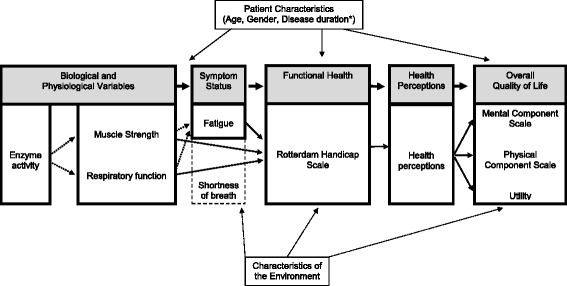
Wilson-Cleary conceptual disease model applied to Pompe disease

Figure 1 presents the final conceptual model for Pompe disease. The conceptual model can be used to predict quality of life by inserting clinical data in the formulas found in the various models (as depicted in the Additional file 1: Figure S1). In this respect, it should be noted that the estimated coefficients from the regression analyses (Table 2) are all surrounded with varying amounts of uncertainty, which is presented in the Additional file 1: Figure S1.

## Discussion

It has often been stated that the common methodology for clinical and cost-effectiveness studies is unsuitable for orphan diseases, due to the limited number of patients [1, 39]. One recurring problem stemming from the low disease frequency is limited statistical power. A disease model can be used when long-term follow-up is unavailable. The concept of a disease model incorporates disease progression based on a combination of clinical plausibility and statistical analysis. Here we present a disease model for Pompe disease. Empirical data was used to test the relationships in this model. The model forms a starting point, to which information about treatment effects can be added in the future.

### Purpose and validity of the model

We set out to describe the natural course of Pompe disease using an application of the Wilson-Cleary model. The appropriate structure of the model is disease dependent. For some disease progression models, classification of disease states is feasible (e.g. cancer, arthritis, multiple sclerosis, heart disease). Pompe disease is characterized by a continuous range of phenotypes, which makes identification of disease states difficult. Furthermore, accurately estimating parameter values for disease states is hampered by small sample sizes. Therefore, we applied the Wilson Cleary conceptual model rather than a model containing health states. Content validity (i.e. the validity of the associations that were modelled) of this model is ensured by the clinical experts that were involved in the development of the model and evidence from the literature. By comparing the results of the final model to findings reported in the literature construct validity can be assessed. Such comparisons reveal that the conceptual model is largely supported by the data since many of the results correspond with findings from previous studies. Disease duration is negatively associated with MRC sum score, in line with earlier findings [21, 35, 36]. The rate of MRC sum score deterioration (0.6 % points per year) is comparable to our previous publications [22, 38]. No significant association between disease duration and FVC was found (*p* = 0.190), in contrast to findings described earlier [20, 35, 36]. However, the relatively small though nonsignificant p-value we found in our analysis may be an indication of limited statistical power, a reduced ability to detect an existing association. Female patients have higher FVC than male patients, which is similar to the findings of our earlier study [20]. Enzyme activity is not associated with the MRC sum score or FVC, which agrees with the findings of earlier studies that did not find any relationship between enzyme activity and severity in adult patients [8, 36]. Being the underlying biological explanation of the disease, enzyme activity is retained in the conceptual model, despite the absence of a statistical significant association with other variables in the conceptual model.

The genetic mutation is the underlying cause of reduced enzyme activity. However, in our population most of the patients have the same mutation. Hence, mutation is left out of the model.

FSS was not associated with MRC sum score or FVC, albeit that FSS was significantly related to MRC at the 10 % level (*p* = 0.070). Our previous studies did not find a relationship between fatigue and muscle strength and pulmonary function for adult patients with Pompe disease. While more severely affected patients (i.e. those who are in a wheelchair and/or ventilator dependent) do report higher FSS scores, almost all patients report fatigue [24, 40]. For example, 71 % of patients who did not use respiratory support and/or wheelchair reported a FSS score of more than 4 (reflecting fatigue), while 59 % had an FSS score of more than 5 (reflecting severe fatigue). Also, even when patients are still in the pre-clinical stage of the disease they can report fatigue.

RHS is significantly affected by MRC sum score and FVC and better performance on these physiological variables are reflected by lower handicap scores. These findings resemble earlier results that showed that severely affected patients (that is, those requiring ambulatory and respiratory support) reported lower scores on RHS than other patients [29]. The significant relationships indicate that functional status is not fully explained by fatigue, but by other problems related to reduced skeletal muscle strength and pulmonary function as well. One of these problems could be shortness of breath, which was absent in the model due to lack of data.

The conceptual model we present here embodies the current state of knowledge on Pompe disease in adult patients. Data that becomes available in the future can be used to fine-tune the relationships in the model. Moreover, new insights in the measurement of respiratory function might lead to the replacement of FVC by maximal inspiratory pressure (MIP) or maximal expiratory pressure (MEP). Inclusion of shortness of breath in new versions of the model might be reconsidered if new insights or analyses become available in future. Similarly, a newer version of the model can include once such data are available.

#### Strengths and applicability of the model

The availability of a (large) cohort of Pompe patients with several outcome measures enabled statistical testing of the conceptual model. Moreover, the dataset is relatively large compared to other orphan diseases, with available data on 79 patients and multiple observations for most patients. Although the current statistical analyses were based on Dutch patients only, there are no indications that the disease processes differ between the Dutch population and patients in other countries. Therefore, the underlying conceptual relationships described in the model will also be valid in other settings. The results described in this paper are also transferable for use in other countries since the measurement techniques (e.g., to describe muscle function) are widely used and not country-specific.

For many other orphan diseases, the number of patients is too small for a single institution or even a single country to assess a drug’s effectiveness. Data from international disease registries can potentially be used to explore relationships between clinically relevant levels of health concepts of an orphan disease. The testing of longitudinal relationships requires systematic follow-up, which might not yet be available in orphan disease registries.

The use of random effects models appears particularly beneficial in orphan diseases since it can compensate for the low number of patients to a certain extent. For this purpose, the availability of multiple observations per patient is indispensable.

#### Treatment

The assessment of a treatment effect is outside the scope of the current study. The analyses were deliberately restricted to untreated patients because we aimed to develop a model that describes the conceptual intercorrelations between the various levels of health concepts for Pompe disease. However, the conceptual model could be seen as a starting point for developing a full cost-effectiveness model to evaluate the cost-effectiveness of enzyme replacement therapy, assuming that the causal pathway of the disease will not change. Enzyme replacement therapy supplements the low levels of enzyme and has been shown to influence various levels of health concepts in the model, i.e. muscle strength and respiratory function, fatigue, functional health and quality of life, presumably through the supplementation of enzyme [13, 22, 40–42]. If the model would in the future be used to estimate effects of ERT on health outcomes, this could be done in several ways. One way is to apply relative risk reductions to the outcomes as predicted by the equations in the model. These risk reductions would reflect the effect of ERT. Another approach is to re-estimate the equations with ERT as a predictor in the regression models. In both cases we would need longitudinal data on enzyme activity to estimate the impact of changes in enzyme activity on changes in health outcomes.

#### Limitations of the study

The number of observations and study period are relatively limited given the slow disease progression seen in Pompe disease. However, these limitations are common for studies of rare chronic diseases. This limitation could be rectified by international studies that collect relevant patient data in a standardized manner with a minimum loss to follow-up. Another limitation of our study was lack of data on shortness of breath, which could therefore not be included in the model.

#### Transferability of the model

This is the first study in which the Wilson-Cleary model is applied to an orphan disease. Although our model is specifically developed to describe and quantify the relationships between specific levels of health concepts of Pompe disease in adult patients, the approach used in this study can also be applied to other Pompe patient populations, i.e. children and infantile patients, or for other orphan diseases. Obviously, it is necessary to adapt the model to ensure that it contains only relevant disease-specific information and knowledge, although parts of the model developed here for Pompe disease in adult patients may still be relevant. To model Mucopolysaccharidosis type II for instance, “physiological factors” might be expressed using joint angle range and mental retardation can be used as a measure for “functional health”. As another example, Duchenne muscular dystrophy might be adequately represented using the current model if a factor for heart performance were to be included. However, in all cases, the application of the Wilson-Cleary model should be studied for each disease separately.

## Conclusions

The Wilson-Cleary health outcomes model is a helpful tool for developing a conceptual model for Pompe disease. The conceptual model provides a comprehensive overview of all aspects of Pompe disease in adults, integrating subjective and objective health outcomes in one model. This model can be useful to clinicians and researchers who want to explore how changes in specific clinical characteristics may affect a patient’s overall health outcomes. In addition, the model can be useful for policy makers who must consider various issues when making implementation and reimbursement decisions about treatments for orphan diseases. The approach used here provides a means to understand the mechanisms that drive changes in health that may occur both during natural course or if a drug is used to treat patients.

## Additional file

Additional file 1: Figure S1. Analyses based on conceptual model Pompe disease (DOCX 41 kb)

## Abbreviations

EQ-5D: Euroqol 5D
ERT: Enzyme replacement therapy
FVC: Forced vital capacity
FSS: Fatigue severity scale
HRQoL: Health related quality of life
IPA: International pompe association
MCS: Mental component scale
MEP: Maximal expiratory pressure
MIP: Maximal inspiratory pressure
MRC: Medical research council
PCS: Physical component scale
RHS: Rotterdam handicap scale
SF-36: Short form 36
SF-6D: Short form 6D
VAS: Visual analogue scale
