# Supplementary material for: A conceptual disease model for adult Pompe disease
Source: Orphanet J Rare Dis. 2015 Sep 15;10:112. doi: 10.1186/s13023-015-0334-6 (PMC4570629; doi:10.1186/s13023-015-0334-6)
Supplement: Additional file 1: Figure S1. — Analyses based on conceptual model Pompe disease (DOCX 41 kb) [file 13023_2015_334_MOESM1_ESM.docx]

**Figure S1 Analyses based on conceptual model Pompe disease**

Physiological variables

Model I. Muscle strength & Model II. Respiratory function

Explanatory variables: Age, Female, Duration*, Enzyme activity

MRC = **83.452** – 0.069 * Age – 0.028 * Female – **0.563** * Duration + 0.370 * Enzyme activity

(5.221) (0.084) (2.250) (0.116) (0.298)

FVC = **70.526** – 0.144 * Age + **17.994** * Female – 0.346 * Duration + 0.431 * Enzyme activity

(11.915) (0.195) (5.549) (0.263) (0.732)

Symptom status

Model III. FSS

Explanatory variables: Age, Female, Duration*, Enzyme activity, MRC, FVC

FSS = **8.737** + 0.010 * Age + 0.307 * Female **– 0.077** * Duration + 0.007 * Enzyme activity – 0.031 * MRC

(1.700) (0.012) (0.352) (0.020) (0.049) (0.017)

– 0.011 * FVC

(0.075)

Functional status

Model IV. RHS

Explanatory variables: Age, Female, Duration*, Enzyme activity, FSS, MRC, FVC

RHS = **16.969** **– 0.096** * Age – 0.957 * Female – 0.052 * Duration – 0.048 * Enzyme activity – **0.578** * FSS

(4.878) (0.033) (0.936) (0.057) (0.131) (0.207)

+ **0.189** * MRC + **0.075** FVC

(0.046) (0.020)

(General Health perceptions)

Model V. VAS

Explanatory variables: Age, Female, Duration*, Enzyme activity, RHS

VAS = -9.313 + 0.126 * Age + 4.673 * Female + **0.623** * Duration + 0.004 * Enzyme activity + **2.030** * RHS

(14.818) (0.144) (3.334) (0.209) (0.482) (0.292)

(Quality of Life)

Model VI. MCS & Model VII. PCS & Model VIII. Utility

Explanatory variables: Age, Female, Duration*, Enzyme activity, VAS

MCS = **49.990** – 0.016 * Age – 1.282 * Female + 0.063 * Duration – 0.359 * Enzyme activity + **0.157** * VAS

(6.760) (0.092) (2.296) (0.135) (0.325) (0.043)

PCS = **24.526** – 0.046 * Age – 0.402 * Female – 0.086 * Duration – 0.069 * Enzyme activity + **0.223** * VAS

(5.168) (0.069) (1.709) (0.102) (0.243) (0.035)

Utility EQ-5D = **0.530** + 0.001 * Age + 0.010 * Female – 0.001 * Duration – 0.006 * Enzyme activity +

(0.105) (0.001) (0.035) (0.002) (0.005)

**0.004** * VAS

(0.001)

* Duration since diagnosis; bold figures represent significant relationships; standard errors of coefficients are indicated in brackets below the coefficient;
